# Cutaneous horn: A rare subtype of juvenile xanthogranuloma

**DOI:** 10.1002/ccr3.2549

**Published:** 2019-12-19

**Authors:** Fariba Iraji, Zakiye Ganjei, Samira Kazemipour

**Affiliations:** ^1^ Dermatology, Skin Diseases and leishmaniasis Research Center Department of Dermatology Isfahan medical school Isfahan University of Medical science Isfahan Iran

**Keywords:** cutaneous horn, histiocytosis, juvenile xanthogranuloma

## Abstract

Because of variability in the JXG shape and the extensive range of a cutaneous horn differential diagnosis, dermatologists should keep this diagnosis in their mind in the time of encountering with infants or children cases of cutaneous horn.

## Graphical abstract

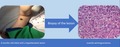

## 1 INTRODUCTION

We included a 6‐month‐old infant with a cutaneous horn in this study. The lesion was described as a long, firm, painless hyperkeratotic nodule with three different colors on his left arm. Pathology and immunohistochemistry indicated juvenile xanthogranuloma (JXG) findings. Dermatologists should consider JXG, during encountering with a cutaneous horn in children.

Juvenile xanthogranuloma (JXG), observed as a benign lesion and usually occurs in infancy or early childhood period,^1^ scientifically is a normolipemic non‐Langerhans cell histiocytosis composed of cells that derived originally from dermal dendrocytes.^2^ JXG has been characterized by two main clinical forms including a micronodular form with multiple small papules on the body upper part and also a macronodular form with one or few larger nodules on the head and trunk.^3^ JXG could present in an extensive shapes and sizes range, as well as locations and distributions,^2^ and has been reported as different clinical appearances including oral mucosal form,^4^, ^5^ hyperkeratotic nodule,^6^ multiple giants,^7^, ^8^ generalized lichenoid eruption,^9^ reticulated maculopapular eruption,^10^ cluster form,^11^ and finally rare cases of cutaneous horn‐like .^12^

This case study presented the case of a 6‐month‐old male infant with a cutaneous horn‐like JXG. This study emphasis is on considering the JXG as one of the differential diagnoses of a cutaneous horn in infants and children.

## 2 CASE REPORT

**Figure 1:**
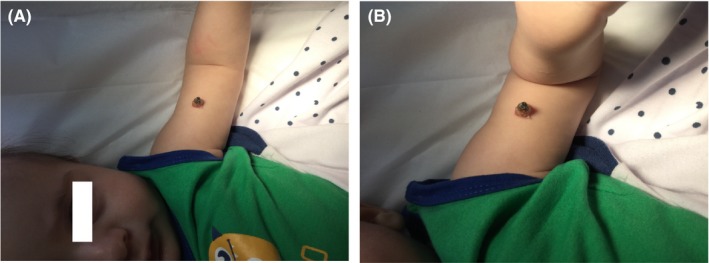
A 6‐month‐old male infant with an elongated hyperkeratotic projection on his left arm approximately 1 cm in width and 2.5 cm in height. The top of the nodule was black, the middle was brown, and the base was yellow. It was firm and painless in palpation

This study investigated the case of a 6‐month‐old healthy male infant, who presented with a long hyperkeratotic projection on his left arm, which was appeared at the age of 4 months as a dark flat lesion that slowly became extended, and also its color has been changed with no history of pain, bleeding, or other symptoms (Figure 1). The infant was born by cesarean section, and no complication associated with his birth was documented.

Physical examinations indicated a long firm and painless hyperkeratotic projection on his left arm, which had approximately 1 cm in width and 2.5 cm in height with a black‐colored top, brown‐colored middle, and yellow‐colored base.

**Figure 2:**
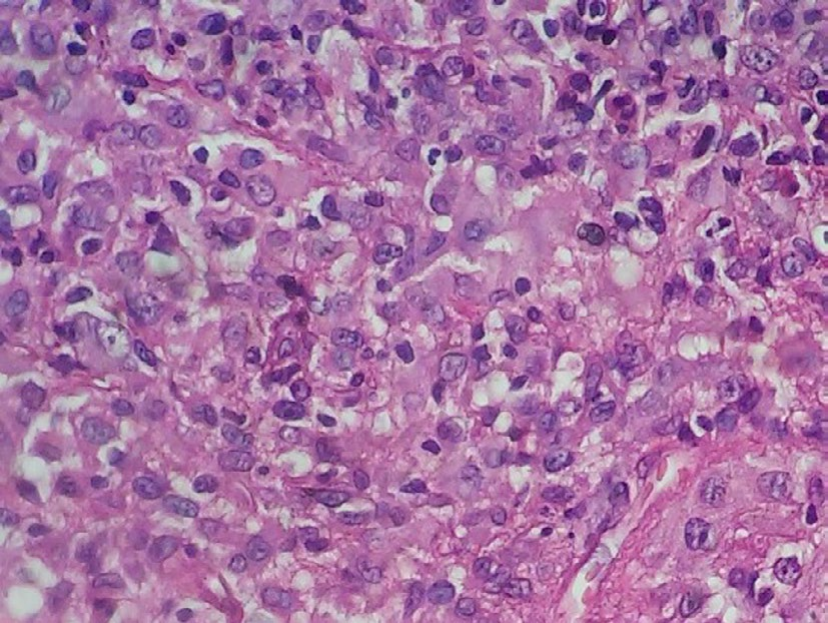
Dense monomorphic foamy histiocytic infiltration and scattered eosinophils but Touton cells were not present

The lesion was excised and a punch biopsy was attained from its base. The hyperkeratotic part, and also the lesion base, was sent for accomplishing histopathologic examination. Although pathologic analysis indicated dense monomorphic foamy histiocytic infiltration, and also scattered eosinophils, there were no Touton giant cells (Figure 2). Since the immunohistochemical staining was positive for CD68/factor XIIa and negative for S100/CD1a/langerin (CD207), the juvenile xanthogranuloma diagnosis was established for the patient.

No abnormality was found in ophthalmologic examination.

## 3 DISCUSSION

A cutaneous horn is a term given to a protrusion from the keratinized material skin that was formed in a horn shape.^13^ It may arise from an extensive epidermal lesions range, which may be benign, premalignant, or malignant. Basal cell papilloma, viral warts, keratoacanthomas, and trichilemmal cysts on one hand, and solar keratosis, Bowen's disease, and SCC, on the other hand, are the benign and premalignant examples, or completely malignant lesions, respectively, and can have a clinical presentation as same as a cutaneous horn.^13^ However, only a few reports about the cutaneous horn in children have been reported in the papers. The differential diagnosis can be varied in this age group. Although molluscum contagiosum,^14^ common wart,^15^ subepidermal calcified nodule,^16^ and pyogenic granuloma^17^ are considered as frequent causes of the horn in infants and children, there also exist unusual causes like JXG in the researches.^12^, ^18^, ^19^

Consequently, JXG indicates great variability in clinical presentation.^1^ Therefore, it is difficult and sometimes impossible for the physician to diagnose this lesion without the help of pathologic examination. Moreover, it is not regular for dermatologists to consider a cutaneous horn as a juvenile xanthogranuloma. To our best of knowledge, there are only three reports accomplished on cutaneous horn‐like JXG in children, up to now,^12^, ^18^, ^19^ and our case study is the fourth.

Since JXG can show numerous clinical presentations and the cutaneous horn has an extensive range of differential diagnosis, dermatologists should keep this diagnosis in their mind at the time of encountering with infants and children cases of cutaneous horn. In addition, careful attention to the JXG lesions clue features like the yellow hue of the lesions could be considered as helpful indicators in order to make an accurate diagnosis.

## CONFLICT OF INTEREST

The authors deny any conflict of interest during the study.

## AUTHOR CONTRIBUTIONS

FI: was responsible for medical care. ZG: interpreted and reviewed the patient data, revised the manuscript, and performed patient follow‐up. SK: performed biopsy, drafted the initial manuscript, and provided photographs.
